# Spatial Copula Model for Imputing Traffic Flow Data from Remote Microwave Sensors

**DOI:** 10.3390/s17102160

**Published:** 2017-09-21

**Authors:** Xiaolei Ma, Sen Luan, Bowen Du, Bin Yu

**Affiliations:** 1 School of Transportation Science and Engineering, Beijing Key Laboratory for Cooperative Vehicle Infrastructure System and Safety Control, Beihang University, Beijing 100191, China; xiaolei@buaa.edu.cn (X.M.); luansenda@buaa.edu.cn (S.L.); yubinyb@buaa.edu.cn (B.Y.); 2 Key Laboratory of Road & Traffic Engineering of the Ministry of Education, Tongji University, 4800 Cao’an Road, Shanghai 201804, China; 3 School of Computer Science and Engineering, the State Key Laboratory of Software Development Environment, Beihang University, Beijing 100191, China

**Keywords:** traffic flow imputation, spatial interpolation, spatial correlation, marginal distribution, copula model

## Abstract

Issues of missing data have become increasingly serious with the rapid increase in usage of traffic sensors. Analyses of the Beijing ring expressway have showed that up to 50% of microwave sensors pose missing values. The imputation of missing traffic data must be urgently solved although a precise solution that cannot be easily achieved due to the significant number of missing portions. In this study, copula-based models are proposed for the spatial interpolation of traffic flow from remote traffic microwave sensors. Most existing interpolation methods only rely on covariance functions to depict spatial correlation and are unsuitable for coping with anomalies due to Gaussian consumption. Copula theory overcomes this issue and provides a connection between the correlation function and the marginal distribution function of traffic flow. To validate copula-based models, a comparison with three kriging methods is conducted. Results indicate that copula-based models outperform kriging methods, especially on roads with irregular traffic patterns. Copula-based models demonstrate significant potential to impute missing data in large-scale transportation networks.

## 1. Introduction

Traffic detection is an important component of intelligent transportation systems. The collected traffic flow information provides the basis for urban traffic planning and management. However, the current traffic flow collection and analysis face two major issues. One issue is missing data, which result from equipment failure and processing errors. Almost 50% of road permanent traffic counts (PTCs) feature missing data [1]. This issue becomes more serious for remote microwave sensor data in China [2]. Another issue corresponds to data sparseness, which results from the low coverage of detectors and requires additional efforts to estimate intermediate traffic conditions between adjacent traffic sensors. These two issues are summarized as incompleteness of traffic flow data. The Texas Transportation Research Institute has reported that the completeness ratio of the data that is archived in transportation management systems increased from 16% to 93% with a data cleansing procedure [3]. Traffic flow can be utilized to calculate traffic parameters such as annual average daily traffic (AADT) and road capacity. Therefore, missing traffic flow must be recovered for use by transportation planners and operators. 

The integrity of traffic data is an important theme that has been discussed for nearly two decades. The principle of *Highway Traffic Monitoring Standards* [4] from the American Society for Testing and Materials Standard Practice and the American Association of State Highway and Transportation Officials Guidelines [5] state that traffic measurements must be raw before they are saved as base data. However, the imputation of traffic data is not necessarily prohibited during analysis. Traffic data with missing values may be the only data available for certain purposes [1]; thus, imputation of traffic flow is necessary for further analysis. For traffic flow data with missing values, traffic management agencies usually retake or impute based on incomplete observed data. Albright [6] emphasized the use of excessive manpower and time to retake data collection from detectors with missing data and mentioned the imputation of missing traffic data as a common countermeasure by many traffic agencies in the United States. 

A series of methods have been proposed to impute missing traffic data. The autoregressive integrated moving average (ARIMA) model [7,8] is often adopted in studies of time-series traffic prediction, where long-term trends, such as regular daily recurrent congestions, can be observed from traffic flow fluctuation. However, ARIMA is more suitable for short-term traffic prediction with stable traffic patterns and may not be applicable for scenarios with large portions of missing data. Gazis and Liu [9] considered the sharing error at two adjacent road links and developed an extended Kalman filter approach for traffic flow estimation. The Bayesian and Markov models rely on prior knowledge to obtain estimated parameters [10,11,12]; these models require substantial historical data as prior knowledge. In the context of big data, machine-learning-based methods have emerged. Neural networks and their variants are proposed by a number of scholars for traffic prediction [13,14,15] and have presented promising prediction results. As representative statistical models, multi-variable-based methods are also widely used in traffic prediction. Multiple or weighted regression models [16,17] are based on kernel functions, which assign different weight values to independent variables. Common independent variables include historical traffic flow, weather information, and land use. Collecting external variables is time consuming and expensive for model construction of large-scale transportation networks. Lam and Xu [18] compared two models, namely, regression and neural networks, to estimate AADT based on short-period counts in Hong Kong, suggesting that neural networks outperform regression methods.

The review of the above literature presented a common feature of treating data collection sites as isolated. This feature cannot easily expand to road networks with high numbers of malfunctioning sensors. Huang et al. [19] pointed out that methods based on historical data will no longer apply when the missing data ratio is high. Missing traffic data can be ideally imputed by using only small valid samples collected from observed sensors. Implementation of this data imputation method can not only reduce economic expenditure of PTC deployment, but can also provide convenience for traffic operators. To achieve this goal, the spatial dependency of traffic flow from adjacent traffic sensors should be incorporated. Spatial interpolation of imputed missing traffic flow data initially captures the spatial dependence of other data collection sites at the same timestamp. Then, missing values are remedied by observed traffic flow data based on spatial dependence. The function that describes spatial dependence is a covariance function based on spatial distance. Kriging is the mainstream spatial interpolation approach based on covariance functions. The fundamental theory of kriging can be found in the works of Cressie [20] and Stein [21]. In the transportation domain, a number of successful studies used Kriging for traffic flow imputation. Wang and Kockelman [22] utilized the Texas highway count data as model input in the Euclidean distance scale and observed that kriging is a promising method and can be applied to a variety of data sets for regression kriging [23]. Zou et al. [24] compared the Euclidean distance and road net distance and demonstrated that the use of distance from road net can perform better in traffic speed interpolation. Shamo et al. [25] compared simple kriging (SK), ordinary kriging (OK), and universal kriging (UK) and discovered that combined with different correlation functions, the same kriging method consistently received suboptimal performance for AADT for 2008. The same researchers also demonstrated that the lack of optimal results for AADT for 2009 and 2010 was caused by data that undermined the assumption of the Gaussian stationary process when using kriging-based methods. The proposed kriging methods are not applicable when spatial correlation is relatively weak, thus coinciding with the results of Zhang et al. [26]. Compared with AADT data, hourly traffic flow data fluctuates more significantly and therefore require a much more advanced method to relax the constraints of the stationary assumption.

This paper therefore proposes copula-based methods for their adaptability to data with high variability or extreme values [27,28]. Copula theory is commonly used in finance time series between stock markets for correlation analysis [29,30,31], and has been recently applied to transportation. For example, Bhat et al. [32] adopted the concept of copula to analyze the relationship between the daily miles of travel of household vehicles and residential neighborhood selection. Sener and Reeder [33] explored the effects of the intense activity of workers on active travel behavior. Zou and Zhang [34] examined the application of copula in a joint model of speed, headway, and vehicle length and proved dependency among these variables. Copula theory combines fitted marginal distribution functions from traffic flow data as a joint distribution function. However, we must firstly analyze spatial dependence to provide the required parameters for the joint distribution function. Finally, we accumulate the probability of the joint distribution function to estimate the missing traffic flow data. With additional support of the marginal distribution function, the copula-based model can more accurately describe spatial dependency than kriging methods [35]. Copula also features variants that adapt to different roads with varying traffic patterns.

In view of the required data conversion in the interpolation process, we currently use Gaussian copula and non-Gaussian methods, such as Chi-square and Student’s t, to construct models and apply them to two expressways in Beijing. According to Kazianka’s work [36], the spatial copula is designed to address the need for researchers to analyze spatial data which are markedly non-Gaussian. They also find Gaussian copula is applied in most applications for computational reasons. Gaussian copula has radial symmetry, implying that either the high or low tail of any distribution has an equal dependence [37]. This assumption may not be applicable for modeling extreme events. To tackle the shortcoming of Gaussian copula, the property of radial asymmetry should be introduced. For example, high values of data generate a stronger spatial dependency than low values. Kazianka and Pliz [38] have proposed to use non-central chi-squared copula to interpolate spatial data. In the transportation domain, non-recurrent congestion (e.g., accidents and adverse weather conditions) may produce heavy traffic flows. Therefore, the non-Gaussian copula is particularly suitable to spatially impute traffic flow data under extreme traffic conditions. This study aims to examine the applicability of different spatial copula methods in dealing with both stationary and extreme traffic data. In addition, kriging methods, such as classical spatial interpolation approaches, are also carried out for comparison. The main contribution of this study is to propose a copula-based spatial interpolation model for imputing missing traffic flow data from remote microwave sensors.

The remaining sections of this paper are organized as follows. In the next section, the interpolation model is constructed based on basic copula theory. Section 3 describes the traffic flow data from remote microwave sensors in Beijing. Section 4 lists parameters from copula-based models and analyzes the interpolation results in comparison with kriging. The final section summarizes conclusions and future work.

## 2. Methodology

### 2.1. General Copula Theory

Copula theory was first proposed by Sklar [39] in 1959 and has been applied actively in the field of statistics. In statistics, copula acts a multivariate or joint distribution function; it combines the joint distribution function with the marginal distribution function of variables [40]. The detailed concept of general copula theory is described as follows.

Sklar’s theory [39] demonstrates that a joint distribution can be decomposed into a set of one-dimensional margin distributions and a copula function. A *P*-dimensional joint distribution function of traffic flow can be synthesized by some marginal distribution functions as follows:

(1) $$F_{p}(Z_{p})=F\left(z_{1},\ldots ,z_{p}\right)=C_{\theta }\left(F_{1}\left(z_{1}\right),\ldots ,F_{p}\left(z_{p}\right)\right)$$

where $Z_{p}$ represents *P*-dimensional traffic flow, and *θ* refers to a parameter set of the copula that controls the spatial dependence of traffic flow. Copula function can be obtained based on Equation (1) via an inversion method [38]. For example, given a known joint distribution *F_c_*($z_{1},z_{2},,,z_{p}$) with continuous marginal distribution function $F_{p}(Z_{p})$, the inversion method can be described as follows:

(2) $$\{\begin{array}{c} u_{i}=F_{i}\left(z_{i}\right),i=1,\ldots ,p\\ C\left(u_{1},\ldots ,u_{p}\right)=F_{c}\left(F_{1}^{-1}\left(u_{1}\right),\ldots ,F_{p}^{-1}\left(u_{p}\right)\right) \end{array}$$

where $u_{i}\in \left[0,1\right]$ and $F_{i}^{-1}$ denote the inverse functions of $F_{i}$.

Copula is essentially a joint distribution function with varying types of probability distributions. This study executes three types of copula, including Gaussian, Chi-square, and Student’s t, to investigate the feasibility of imputing missing traffic flow data on expressways.

### 2.2. Spatial Copula Modelling

The purpose of this section is to construct a function between the missing value and the adjacent known observations as a form of ${\hat{z}}_{0}(x_{0},y_{0})=f(z_{1}(x_{1},y_{1}),\ldots ,z_{n}(x_{n},y_{n}))$, where ${\hat{z}}_{0}$ is the interpolated traffic flow at location $\left(x_{0},y_{0}\right)$ and $z_{i}(x_{i},y_{i})$ represents the observed values at adjacent locations. The main steps of the model construction are described as follows. We first used the actual observed traffic flow to fit out the optimal correlation function and marginal distribution function. Then, the parameters of these two functions were estimated using the canonical maximum likelihood (CML) method. Next, copula established a conditional probability function of the missing value based on weighted observed data processed by correlation function. Finally, the marginal distributions were joined by the copula function, then the conditional probability function was integrated to obtain predicted values of missing traffic flow data.

#### 2.2.1. Correlation Function Fitting

Step 1. Calculate the Euclidean distance of any two microwave sensors, which can be represented in matrix form as follows:

(3) $$D=\left(\begin{array}{ccccc} 0 & d_{1,2} & \cdots & d_{1,n-1} & d_{1,n}\\ d_{2,1} & 0 & & & d_{2,n}\\ \vdots & & \ddots & & \vdots \\ d_{n-1,1} & & & 0 & d_{n-1,n}\\ d_{n,1} & d_{n,2} & \cdots & d_{n,n-1} & 0 \end{array}\right)$$

where $d_{i,j}=d_{j,i}=\sqrt{{\left(x_{i}-x_{j}\right)}^{2}+{\left(y_{i}-y_{j}\right)}^{2}}$, and unit of d measures 0.5 km.

Step 2. Set up lag distances h ($h_{0},h_{1},h_{2},h_{3},h_{4}，\cdots ,h_{m}$) and $h_{0}=0$ by default. The value of $h_{i}$ is a dynamic value based on the location of the actual microwave sensor. The principle of assigning values to $h_{i}$ ensures that 10–20 elements from D lie in the range of ($h_{i},h_{i+1}$). Considering ($h_{0},h_{1}$) as an example, we can set $d_{i,j}$ = 1 if $h_{0}$ < $d_{i,j}$ < $h_{1}$ as true; otherwise, $d_{i,j}$ = 0. To simplify the following formulas, we suppose that four elements from D satisfy $h_{0}$ < $d_{i,j}$ < $h_{1}$. Thus, matrix D becomes sparse as follows:

(4) $$D_{h_{0}<d<h_{1}}=\left(\begin{array}{ccccc} 0 & 0 & \cdots & 1 & 0\\ 0 & 0 & & & 1\\ \vdots & & \ddots & & \vdots \\ 1 & & & 0 & 0\\ 0 & 1 & \cdots & 0 & 0 \end{array}\right)$$

Step 3. Calculate the difference between $z_{i}$ and $z_{j}$ (i,j=1,2,3,…) using $z_{i,j}$ = $\left|z_{i}-z_{j}\right|$. z represents traffic flow, where $z_{i}$ and $z_{j}$ are obtained from different microwave sensors.

(5) $$D_{z}=\left(\begin{array}{ccccc} 0 & z_{1,2} & \cdots & z_{1,n-1} & z_{1,n}\\ z_{2,1} & 0 & & & z_{2,n}\\ \vdots & & \ddots & & \vdots \\ z_{n-1,1} & & & 0 & z_{n-1,n}\\ z_{n,1} & z_{n,2} & \cdots & z_{n,n-1} & 0 \end{array}\right)$$

Then, [$Z_{1,n-1}$ $Z_{2,n}$ $Z_{n-1,1}$ $Z_{n,2}$] are selected from matrix $D_{z}$, and [$d_{1,n-1}$ $d_{2,n}$ $d_{n-1,1}$ $d_{n,2}$] are selected from D according to the position where “1” appears in the sparse matrix $D_{h_{0}<d<h_{1}}$.

Step 4. The average values of traffic flow and distance are calculated to avoid extreme data or outliers for describing spatial dependence in the following Equation (8).

(6) $$Z_{h_{1}}=(Z_{1,n-1}+Z_{2,n}+Z_{n-1,1}+Z_{n,2})/4$$

(7) $$H_{h_{1}}=(d_{1,n-1}+d_{2,n}+d_{n-1,1}+d_{n,2})/4$$

Repeat Steps 2–4 with different ($h_{i},h_{{}_{i+1}}$). Then, $Z_{m}$ = [$Z_{h_{1}}$ $Z_{h_{2}}$ $Z_{h_{3}}$ … $Z_{h_{m}}$] and $H_{m}$= [$H_{h_{1}}$ $H_{h_{2}}$ $H_{h_{3}}$ … $H_{h_{m}}$] are obtained. Values of ***m*** should not be extremely high to prevent excessive computing burden.

Step 5. Two parameters must be calculated for optimal correlation function fitting.

(8) $$\{\begin{array}{c} a=\sqrt{{\left(X_{\max}-X_{\min}\right)}^{2}+{\left(Y_{\max}-Y_{\min}\right)}^{2}}\\ l=\frac{\max(Z_{m})+\mathrm{mid}(Z_{m})}{2}\\ c_{0}=\frac{\min(Z_{m})}{l} \end{array}$$

where max(), min(), and mid() represent maximum, minimum, and median values in the data set, respectively.

Step 6. Let $\delta =[c_{0},a,\delta_{3}]$. Then, $\mathrm{cov}(H_{m},\delta )$ listed in Table 1 represents the correlation function employed in this paper.

In the four functions in Table 1, δ(1) is referred to as nugget, which means measurement error in practice. The correlation function indicates that spatial correlation decreases with creasing distance. The common feature of these four functions is that the corresponding value of each function ceases to change when distance reaches δ(2).Thus, spatial correlation eventually disappears within increasing distance.

Fitting the correlation function identifies a specific correlation function in Table 1, and this function achieves minimum mse. Equation (9) explains this process in detail. The correlation function that produces minimum mse serves as the optimal function.

(9) $$\{\begin{array}{c} l=(\max(Z_{m})+\min(Z_{m}))/2\\ mse=\sum_{i=1}^{m}\frac{m{\left(l(\mathrm{cov}(0,\delta )-\mathrm{cov}(H_{h_{i}}))-Z_{h_{i}}\right)}^{2}}{2\sum_{i=1}^{m}l^{2}{\left(\mathrm{cov}(0,\delta )-\mathrm{cov}(H_{h_{i}})\right)}^{2}} \end{array}$$

#### 2.2.2. Marginal Distribution Fitting

The determination of the marginal distribution function is also conducted by fitting. This study provides five default priori distribution functions, including normal, generalized extreme value (GEV), gamma, log-normal (Logn), and Box–Cox distribution.

u and σ are common parameters that depict the majority of distribution functions, but several still available distribution functions use more parameters. k represents a shape parameter of GEV distribution, and a,b are found in gamma distribution. Except for u and σ, Box–Cox distribution features an additional parameter λ. Thus, in view of the diversity of parameters, we used the parameter set ψ{u,σ,k,λ,a,b,…} for simplicity. These parameters can be calculated based on an a priori distribution function and actual traffic flow data.

Then, these obtained parameters were used to calculate the probability of traffic flow under different apriori distribution functions. Next, the probabilities were summed up to ensure that each distribution corresponds to an accumulated probability value. The optimal distribution function produces maximum *P* based on Equation (10).

(10) $$P=\sum_{i=1}^{n}\log(f_{m}(z_{i},\psi ))$$

#### 2.2.3. Parameter Estimation

The exact maximum likelihood method [41] and inference functions for the margins method [42] are two widely used parameter estimation methods. However, these methods need to specify the marginal distribution type in advance. Therefore, in this study, the CML method was selected due to the uncertainty of the actual correlation function and the distribution function of traffic flow.

First, the raw traffic flow must be converted to a normalized value between 0 and 1 of s specified copula. This conversion consists of two steps, as shown in Equation (11). Traffic flow obtains the corresponding cumulative probability by means of cumulative distribution function (cdf). Then, cumulative probability is converted to the normalized data by the inverse of copula function, as follows:

(11) $$\{\begin{array}{c} P_{i}^{Z}=F_{m}(Z_{i},u,\sigma^{2})\\ z_{i}^{c}=F_{c}^{-1}(P_{i}^{Z}) \end{array}$$

where u and $\sigma^{2}$ refer to the mean and variance of traffic flow, respectively; $F_{c}$ corresponds to a copula function; and $Z_{i}^{c}$ denotes the converted normalized value.

Let Θ=(ψ,δ) denote all model parameters that must be estimated. Thus, based on CML method, Equation (12) is given as the likelihood function. This likelihood function consists of a marginal distribution and a copula function. This two-stage estimator is selected to be computationally tractable, and a one-stage estimator is only proposed and implemented on bivariate data [43].

(12) $$L(\Theta ;\;z)\;=\;c_{\Theta }(F_{m}(z_{1}),\ldots ,F_{m}(z_{n}))\prod_{i=1}^{n}f_{m}(z_{i})$$

where $C_{\Theta }$ denotes copula density function, $F_{m}$ is the cdf and $f_{m}$ represents the pdf.

#### 2.2.4. Spatial Copula Interpolation

Spatial interpolation indicates that the predicted value of traffic flow is generated at a given position based on these estimated parameters. Interpolation accurately describes the relationship between sensors with missing traffic flow (defined as the predictor) and the surrounding observed microwave sensors (defined as referenced points).

Thus, the first step is to calculate weights of the referenced points. Let $z(x_{0},y_{0})$ be a predictor with coordinate $\left(x_{0},y_{0}\right)$; select $N^{*}$ referenced points, and calculate their Euclidean distance vector ($d^{*}$) from the predictor. Then, calculate the distance between each two referenced points and express them in the form of Equation (3), denoted as $D_{ref}$. Therefore, the weight values of the referenced points can be calculated as follows:

(13) $$w^{*}=\frac{\mathrm{cov}(d^{*},\delta )}{\mathrm{cov}(D_{ref},\delta )}$$

where $d^{*}$ and $w^{*}$ corresponds to $N^{*}$ dimensional vectors; and $D_{ref}$ is an $N^{*}\times N^{*}$ matrix. As time evolves, traffic flow from the upstream sensor may reach the downstream sensor and form a strong spatial relationship [44]. Thus, in general, the predictor features a strong relationship with its adjacent referenced points. Thus, the value of $N^{*}$ should not be extremely high. High values of $N^{*}$ may incur excessive computational burdens. In this paper, the value of $N^{*}$ does not exceed 15.

The second step is constructing the conditional pdf of spatial interpolation for the predictor at an unmeasured location $\left(x_{0},y_{0}\right)$. The interpolation of pdf is as follows:

(14) $$P(z_{0}|\hat{\Theta },z_{ref}^{c})=c_{\hat{\Theta }}(F_{c}(z_{0})|z_{ref}^{c})f_{c}(z_{0})$$

where $\hat{\Theta }$ refers to the estimation of Θ, and $Z_{ref}^{c}$ represents converted normalized data of the referenced points. $f_{c}$ and $F_{c}$ correspond to the pdf and cdf of the copula function, respectively, and $c_{\Theta }(\cdot |\;z_{ref}^{c})$ denotes the conditional copula density function. Finally, the interpolation of predictors is conducted by numerical integration as follows.

(15) $$u=w^{*}f_{c}(z_{ref}^{c})$$

(16) $${\hat{z}}_{0}=\int_{0}^{1}F_{c}^{-1}(u)c_{\hat{\Theta }}(u|z_{ref}^{c})du$$

## 3. Data Source

**Figure 1:**
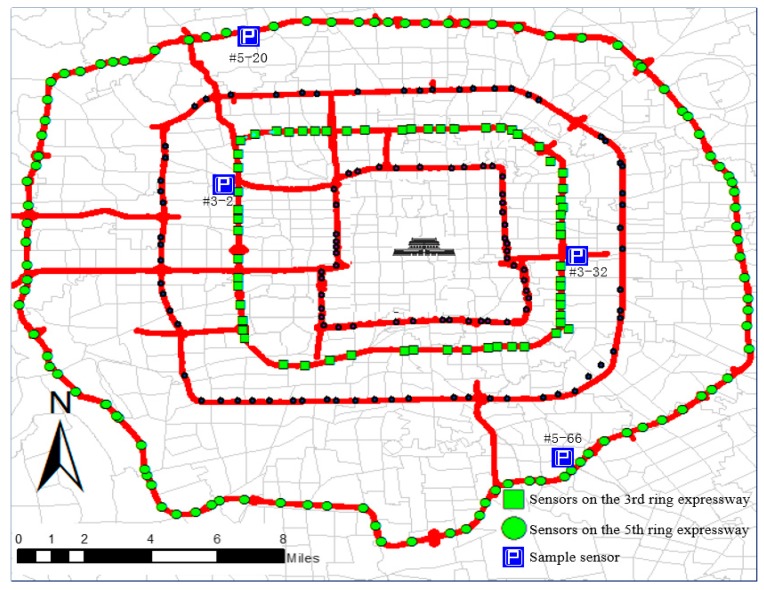
Deployment of microwave sensors on Beijing ring expressways.

The data used in this study were collected from 454 remote microwave sensors deployed at two ring expressways in Beijing on June 1 (Children’s day), June 4 (a typical weekday), and June 7 (a typical weekend), 2015. The key information mainly includes the detector locations in latitude and longitude, timestamp, traffic flow, speed, and occupancy. The frequency of data updating spanned 2 min. The microwave sensors shown in Figure 1 are deployed almost evenly on the ring expressway, but several detectors are sparsely deployed in certain areas. Although microwave sensors are more efficient and reliable than traditional loop detectors, they still feature erroneous and missing data issues.

Table 2 summarizes the data quality for all sensors located in the 3rd and 5th ring expressways at three timestamps. The “difference” field represents the positive difference between the number of valid sensors and the total number of deployed sensors. The “missing percentage” field indicates the missing data ratio, which ranges from 27% to 82%. The average missing percentage totals 48.2%, accounting for half of the malfunctioning sensors.

**Figure 2:**
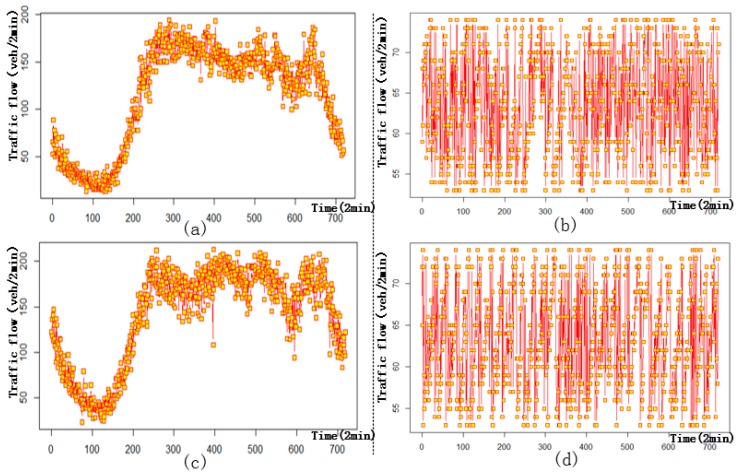
Time series of traffic flow on 1 June 2015. (**a**) #3-2 sensors on the 3rd ring expressway; (**b**) #5-20 sensor on the 5th ring expressway; (**c**) #3-32 sensors on the 3rd ring expressway; (**d**) #5-66 sensor on the 5th ring expressway.

The images in Figure 2 correspond to the four sample sensors marked in Figure 1, and Figure 2a,b displays time series of traffic flow at two random places located on the 3rd and 5th ring expressway, respectively. We only present the time series from June 1 because the daily trends are similar on each weekday or weekend. Figure 2a shows irregular fluctuations of traffic flow on the 3rd ring expressway. Traffic flow from 8:00 (timestamp equal to 240) to 22:00 (timestamp equal to 660) increases, then gradually decreases from 22:00 to 4:00 (timestamp equal to 120) on the next day, before finally increasing until 8:00. As shown in Figure 2b, traffic flow on the 5th ring expressway remains between 50 and 80 vehicles every 2 min throughout the entire day.

Similar traffic flow patterns can be observed at the two sensors located on the same expressway. Figure 2 shows the number of similarities between two random detectors on the 3rd and 5th ring expressways, indicating strong spatial similarities. This factor lays the foundation for the following spatial interpolation.

**Figure 3:**
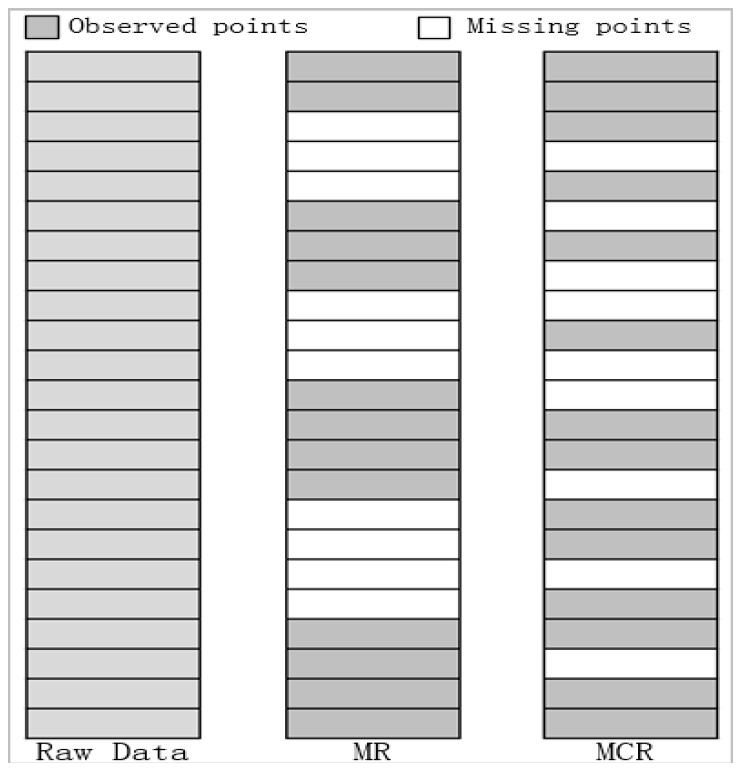
The schematic diagram of the MR and MCR.

To test the effectiveness of the proposed algorithm, we randomly extract 50% of the observed data as the training data set, and the remaining 50% is used as the test data set. The coordinates and corresponding traffic flow data derived from discrete sample detectors served as inputs for modeling. In addition, in order to fully validate the proposed model, we added the data sets from the sensors on the 3rd ring expressway at 8:00 (timestamp equal to 240) on 4 June 2015 and 17:00 (timestamp equal to 510) on 7 June 2015. Each data set has two missing types, including missing at random (MR), and missing completely at random (MCR) according to Qu et al. [45]. Figure 3 shows a schematic diagram of the MR and MCR types.

## 4. Case Study

The parameters of the marginal distribution and correlation function of the 3rd and 5th ring expressways in Beijing were initially computed in this section. Then, the results were calculated using the MATLAB toolbox “Spatial Copula” based on Kazianka’s study [36] and were utilized to compare with kriging methods.

### 4.1. Model Parameters

Table 3 demonstrates the parameter estimation results of the copula model for the 3rd and 5th ring expressways, where $\hat{\psi }$ represents the set of estimated values of the marginal distribution function, and $\hat{\delta }$ is the set of estimated parameters of the spatial correlation function. Traffic flow from the 3rd ring expressway belongs to the GEV distribution with Matern-type spatial dependency, whereas a Logn distribution and a spherical spatial correlation describe the situation of the 5th ring expressway. Therefore, the traffic state in the 3rd ring expressway is more complex than that in the 5th ring expressway, agreeing with the results shown in Figure 2.

In the distribution function, $\hat{u}$ usually represents the location parameter, and $\hat{\sigma }$ generally corresponds to scale parameter. $\hat{k}$ indicates the shape parameter for GEV distribution. The correlation types in Table 1 indicate different dependencies of spatial relationship measures. As the distance between sensors increases to a certain threshold, spatial correlation will almost reach zero in different trends. In addition, $\hat{\delta }(3)$ in the Matern correlation function was given an initial value of 0.5.

### 4.2. Results and Comparison

#### 4.2.1. The 3rd and 5th Ring Expressway with MCR

In this section, we establish three different interpolation models based on copula theory, and spatial interpolation is described with a data loss rate of 50% for an MCR type. We compare the established models with three kriging methods to verify the optimal model using mean absolute percentage error (MAPE) as the comparison indicator. The two groups of datasets from different timestamps are used to evaluate model performance. The selected testing scenarios include morning peak hour (8 AM in the morning) and noon (12:00 PM). These tests aim to determine whether time of day influences model accuracy.

The three types of copula models comprise Gaussian, Chi-square, and Student’s t, as shown in Table 4. We can control the number of referenced points to obtain the best interpolation accuracy. In this study, the number of referenced points, $N^{*}$, was set to 2, 6, 10, and 14. From the selection rule of referenced points described in Section 2.2.1, when the number of referenced points increases, the space under consideration and distance between predictors and referenced points on the same ring expressway also increase. We recommend that the distance should not be extremely high in view of the characteristics of the correlation function.

Table 4 shows the interpolation precision of different copula models based on traffic flow data at morning peak. On the 3rd ring expressway, all three copula models achieved high interpolation accuracy when $N^{*}$ equals 2, and the Gaussian copula and Chi-square copula obtain the optimal MAPE value of 0.2379. This case occurs on the 5th ring expressway when $N^{*}$ equals 10, and the Chi-square copula shows the best performance for interpolation MAPE at 0.0839. When the three copula models are compared at the same $N^{*}$ value, the Chi-square copula usually performed a little better than Gaussian and Student’s t. Thus, the Chi-square copula captures the spatial dependence of traffic flow more efficiently. This result is consistent with the findings of Kazianka [36] in the field of environmental science.

The kriging method is favored among spatial interpolation approaches. To verify the performance of the copula models, we compare the copula-based models with kriging methods. Table 5 presents the results of MAPE. The kriging method relies on an a priori correlation function to describe a spatially-dependent trend. In this paper, the correlation function is used in both the exponential (Exp) and spherical (Sph) types. The MAPE of the copula in Table 5 is obtained from the corresponding optimal value in Table 4. The copula and kriging methods obtain high accuracy on the 5th ring with marginal difference. However, the kriging methods do not perform better than the copula model on the 3rd ring expressway. MAPE reaches 0.42 when UK is applied. However, these interpolation results are not accepted by traffic management agencies. The most evident point is that interpolation accuracy on the 5th ring expressway has been superior to that on the 3rd ring expressway. This phenomenon possibly results from the large variability in traffic flow on the 3rd ring expressway during the morning peak period.

To examine how the performance of spatial copula imputation varies at different times of the day, we performed interpolation again based on the data set at noon (timestamp of 360). The data in Table 6 and Table 4 are generally similar except for the $N^{*}$ value at the time of obtaining the best interpolation value. The Gaussian copula perform better on the 3rd ring expressway, whereas the Chi-square copula still outperforms the others on the 5th ring expressway.

Table 7 presents similar results as Table 5. The prediction accuracy of the 5th ring expressway remains better than the 3rd ring expressway regardless of the method used. The performance of the kriging methods, except for UK on the 3rd ring expressway, declined compared with the results in Table 5, indicating that UK remains the least applicable method for this study, whereas copula-based methods are slightly superior.

The data sets at two different timestamps are compared to verify the time-sensitiveness of the performance of copula models. These results also demonstrate the superiority of the copula compared with the kriging methods, especially for the 3rd ring expressway with various traffic patterns. Comparison of the data sets from morning peak and noon did not yield significantly different results. Thus, the proposed spatial copula interpolation model in this study is almost insensitive to timestamp changes.

In addition, the number of $N^{*}$ affects the computational complexity, as mentioned in Section 2.2.4. We completed the spatial imputation of the 50% missing ratio by setting $N^{*}$ as 14 on a 4-core i5 processor with 8 GB RAM. The Gaussian copula needed 4.42 s, the Student copula needed 9.68 s, and the Chi2 copula needed 16.54 s. The kriging methods took approximately 0.72 s under the same conditions due to the simple calculation processes. The computational burdens of spatial copula interpolation were mainly produced by the processes of the covariance matrix inversion and the probability integral. These processes can be accelerated by using the Parallel MATLAB Toolbox named ‘parfor’ [36].

#### 4.2.2. The 3rd Ring Expressway with MCR and MR

In this section, we only test the model performance on data sets from the 3rd ring expressway, because the traffic flow data on the 5th ring expressway are more spatially stable and relatively easier to impute. The data for the 3rd ring expressway were taken from the morning peak of a weekday (4 June 2015) and the evening peak of a weekend (7 June 2015). On the basis of the MCR data type tested in Section 4.2.1., the verification of the MR type was also conducted.

Table 8 and Table 9 shows the MAPE and root mean squared error (RMSE) values of copula and kriging with MR and MCR types at 9:00 on 4 June 2015. We found that the MAPE and RMSE in the kriging methods remained at around 0.25 and 0.30. The copula model was not significantly better than kriging with the MCR type, but for the MR type, the accuracy of the copula increased by 10%.

Similarly, we performed the model comparison between the copula- and kriging-based methods for 17:00 on 7 June 2015. For both the MR and MCR types shown in Table 10 and Table 11, the copula significantly outperformed the kriging methods in terms of MAPE and RMSE. This may be because the traffic flow during evening peak hour on the weekend is more random than weekday evening peak traffic. However, for the copula model, its performance was not greatly affected by the unstable traffic flow due to non-Gaussian copula’s capability to model extreme data; it was thus superior to the kriging methods for both missing types of data.

In overall, the copula model yielded a more substantial improvement over the kriging methods for the MR type than for the MCR type. This fact can be seen from Figure 3, which shows that MR exhibits a large missing region at random road segments, while MCR uniformly generates the location of missing data on the entire expressway, leading to a number of small missing regions. It is more difficult for the MR type to impute traffic flow data for continuous missing regions since there is no adjacent reference data for spatial interpolation. The proposed copula model still maintains a certain degree of robustness, and thus is more suitable for non-stationary and continuous spatial missing traffic data than the kriging methods.

## 5. Conclusions

This paper proposes three copula-based models for the interpolation of missing traffic flow data from remote microwave sensors. This spatial interpolation method analyzes the spatial dependency of traffic flow from a spatial perspective and predicts missing values based on observed traffic flow at other spatial locations. The entire model can be divided into two parts, namely, spatial analysis and spatial interpolation. In the first part, a correlation function was employed to describe the spatial structure of traffic flow, and marginal distribution was used to fit the trend of traffic flow. In the second part, a connection was established between the predictor and the adjacent referenced points using copula functions. This connection provided the basis for spatial interpolation. To evaluate the performance of the proposed copula-based models, SK, OK, and UK were implemented for comparison. Comparison was carried out on two data sets with different timestamps at a 50% missing rate from the ring expressway in Beijing. The results from the two approaches indicate that the copula-based models are more effective than kriging methods, especially on roads, such as the 3rd ring expressway, with complex traffic conditions. The results for the data sets observed from different timestamps showed no significant difference, indicating that copula-based models are insensitive to the effects of temporal changes. Experiments for different missing data types proved that the copula-based models are significantly superior to kriging methods for the MR type, and is a robust way to deal with continuously missing data. 

The proposed model can be further conducted using several approaches in future work. Although the model is applicable to different times of day, the effect of temporal covariance was still not considered. Thus, using traffic flow from different timestamps to model a spatial–temporal structure presents an interesting work proposal [46]. In this way, Yang et al. [47] proposed a sparse representation-based method for spatial–temporal correlation mining to predict city-scale traffic flows. They found that the spatial context can spread out very far. Similarly, Ermagun et al. [48] developed a data de-trending algorithm to evaluate the spatial correlation between both competitive and complementary links in a grid-like traffic network in Minneapolis, USA. They found that a strong negative correlation happens in rush hours, while a positive correlation occurs between upstream and downstream links. The model is currently applied to only one-dimensional road segments instead of entire road networks with freeways and arterial roads. Potential research should enhance the model architecture for a two-dimensional, network-wide spatial interpolation of missing traffic data. In view of the interpolation ability of copula-based models, identifying optimal sensor deployment locations would be another meaningful study.

## Figures and Tables

**Table 1:** Expressions for correlation functions.

Correlation Function	Expression
Gaussian	$\mathrm{cov}(h,\delta )=\left(1-\delta \left(1\right)\right)e^{\frac{-h^{2}}{\delta {\left(2\right)}^{2}}}$
Exponential	$\mathrm{cov}(h,\delta )=\left(1-\delta \left(1\right)\right)e^{\frac{-h}{\delta {\left(2\right)}^{2}}}$
Spherical	$\mathrm{cov}(h,\delta )=\left(1-\delta \left(1\right)\right)\left(1-\frac{3h}{2\delta \left(2\right)}+\frac{h^{3}}{2\delta {\left(2\right)}^{3}}\right)$
Matern	$\mathrm{cov}(h,\delta )=\delta (1)I(h=0)+K_{\delta (3)}\left(\frac{1-\delta (1)}{2^{\delta (3)-1}\Gamma (\delta (3))}\right){\left(\frac{2\delta {\left(3\right)}^{0.5}}{\delta (2)}\right)}^{\delta (3)}$

**Table 2:** Situations of missing traffic data during peak timestamps.

Timestamp	3rd Ring Expressway		5th Ring Expressway	
	Difference	Missing Percentage	Difference	Missing Percentage
8:00	32/67	53%	39/212	82%
8:30	49/67	27%	152/212	28%
9:00	79/67	invalid	102/212	52%

**Table 3:** Estimates of copula model parameters.

Road Name	Margin	Correl.	$\hat{\psi }$	$\hat{\delta }$
3rd ring expressway	**GEV**	Matern	$\hat{u}$ = 109.2332, $\hat{\sigma }$ = 34.0505, $\hat{k}$ = −0.4501	0.0774, 0.4484, 10
5th ring expressway	**Logn**	Spherical	$\hat{u}$ = 4.1455, $\hat{\sigma }$ = 0.0983	0.9553, 117.5820

**Table 4:** Performance of copula models based on MAPE at 8:00 (timestamp = 240) on 1 June 2015.

Road Name	Copula Model	MAPE and Optimal Numbers of Reference Points			
		$N^{*}$ = 2	$N^{*}$ = 6	$N^{*}$ = 10	$N^{*}$ = 14
3rd ring expressway	Gaussian	**0.2397**	0.2667	0.2685	0.2569
	Chi-square	**0.2379**	0.2472	0.2478	0.2448
	Student T	**0.2389**	0.2600	0.2629	0.3148
5th ring expressway	Gaussian	0.0931	0.0854	**0.0847**	0.0864
	Chi-square	0.0924	0.0857	**0.0839**	0.0864
	Student T	0.0928	0.0855	**0.0847**	0.0883

Note: Numbers in boldface indicate the best results for each model.

**Table 5:** Comparison between copula and kriging based on MAPE at 8:00 (timestamp = 240) on 1 June 2015.

Road Name	Prediction Performance for Different Techniques						
	SK		OK		UK		Copula
	Exp	Sph	Exp	Sph	Exp	Sph	
3rd ring expressway	0.2448	0.2490	0.2492	0.2482	0.4190	0.4201	**0.2379**
5th ring expressway	0.0898	0.0895	0.0895	0.0894	0.1006	0.1001	**0.0839**

Note: Numbers in boldface indicate the best results for each model.

**Table 6:** Performance of copula models based on MAPE at12:00 (timestamp = 360) on 1 June 2015.

Road Name	Copula Model	MAPE and Optimal Numbers of Referenced Points			
		$N^{*}$ = 2	$N^{*}$ = 6	$N^{*}$ = 10	$N^{*}$ = 14
3rd ring expressway	Gaussian	0.2157	0.2095	**0.2026**	0.2058
	Chi-square	0.2035	0.1994	**0.1983**	0.2006
	Student T	**0.2243**	0.2246	0.2685	0.2724
5th ring expressway	Gaussian	0.0959	0.0816	**0.0806**	0.0810
	Chi-square	0.0960	**0.0836**	0.0857	0.0857
	Student T	0.0952	0.0833	**0.0823**	0.0920

Note: Numbers in boldface indicate the best results for each model.

**Table 7:** Comparison between copula and kriging based on MAPE at 12:00 (timestamp = 360) on 1 June 2015.

Road Name	Prediction Performance for Different Techniques						
	SK		OK		UK		Copula
	Exp	Sph	Exp	Sph	Exp	Sph	
3rd ring expressway	0.2875	0.2779	0.3038	0.2972	0.3037	0.2925	**0.1983**
5th ring expressway	0.0920	0.0898	0.0919	0.0899	0.0917	0.0896	**0.0806**

Note: Numbers in boldface indicate the best results for each model.

**Table 8:** Comparison between copula and kriging with MR at 9:00 (timestamp = 270) on 4 June 2015.

Road Name		Prediction Performance for Different Techniques						
		SK		OK		UK		Copula
		Exp	Sph	Exp	Sph	Exp	Sph	
3rd ring expressway	MAPE	0.2657	0.2626	0.2396	0.2466	0.2249	0.2274	**0.1530**
	RMSE	31.0720	30.5671	28.9345	29.0480	25.5226	25.9872	**18.3254**

**Table 9:** Comparison between copula and kriging with MCR at 9:00 (timestamp = 270) 4 on June 2015.

Road Name		Prediction Performance for Different Techniques						
		SK		OK		UK		Copula
		Exp	Sph	Exp	Sph	Exp	Sph	
3rd ring expressway	MAPE	0.2720	0.2931	0.2381	0.2478	0.2274	0.2287	**0.2204**
	RMSE	36.5633	37.7494	33.5713	34.3998	37.7863	38.4027	**33.2352**

**Table 10:** Comparison between copula and kriging with MR at 17:00 on 7 June 2015.

Road Name		Prediction Performance for Different Techniques						
		SK		OK		UK		Copula
		Exp	Sph	Exp	Sph	Exp	Sph	
3rd ring expressway	MAPE	0.4208	0.3223	0.4456	0.3835	0.3424	0.3106	**0.2635**
	RMSE	34.8288	34.1639	42.3878	42.8080	37.7154	39.4146	**30.2329**

**Table 11:** Comparison between copula and kriging with MCR at 17:00 on 7 June 2015.

Road Name		Prediction Performance for Different Techniques						
		SK		OK		UK		Copula
		Exp	Sph	Exp	Sph	Exp	Sph	
3rd ring expressway	MAPE	0.3393	0.3224	0.3782	0.3749	0.3805	0.3762	**0.2989**
	RMSE	33.2994	34.0597	39.1025	40.3054	39.6647	39.6647	**35.6846**
